# The effect of large follicle puncture and aspiration on the outcomes of IVF-ET in patients with asynchronized follicles under the long GnRH-a protocol: a retrospective cohort study

**DOI:** 10.1186/s12884-023-05397-9

**Published:** 2023-01-25

**Authors:** Yixuan Wang, Conghui Pang, Haicui Wu, Chaofeng Wei, Yi Yu, Xin Xin, Fang Lian

**Affiliations:** 1 Shandong University of Traditional Chinese Medicine, Jinan, China; 2 Affiliated Hospital of Shandong University of Traditional Chinese Medicine, Jinan, China

**Keywords:** Unsynchronized follicular development, Large follicle puncture and aspiration, IVF-ET, Long-term GnRH-a protocol, Controlled ovarian hyperstimulation

## Abstract

**Objective:**

This retrospective study aimed to explore whether puncturing and aspirating asynchronized large follicles during long GnRH-a protocol COH impacted IVF-ET outcomes.

**Methods:**

A total of 180 patients with asynchronized follicles during long GnRH-a protocol COH were retrospectively analyzed. They were divided into a puncture group, Group 1 (*n* = 81), and a non-puncture group, Group 2 (*n* = 99), according to whether puncture and aspiration were performed on the prematurely developing large follicles. The data of the selected patients were statistically analyzed to assess the effect of large follicle puncture and aspiration during ovulation induction on the final pregnancy results. In addition, we tentatively divided these 180 patients into either Group A (DF ≤ 14 mm) or Group B (DF > 14 mm) according to whether the diameter of the dominant large follicles (DF) exceeded 14 mm at the time of appearance. These two groups were then further divided into four subgroups: Subgroup A1 (DF ≤ 14 mm, patients underwent large follicle puncture), Subgroup A2 (DF ≤ 14 mm, patients did not undergo large follicle puncture), Subgroup B1 (DF > 14 mm, patients underwent large follicle puncture), and Subgroup B2 (DF > 14 mm, patients did not undergo large follicle puncture) based on whether large follicle puncture and aspiration were performed or not, aiming to compare the effects of large follicle puncture and aspiration on the clinical outcomes of patients with dominant large follicles at different time points.

**Results:**

Group 1 exhibited significantly higher oocyte maturation rate (92.3% vs. 88.9%, *P* = 0.009) and high-quality embryo rate (75.2% vs. 65.7%, *P* = 0.007) compared with Group 2. No differences were observed in the number of oocytes retrieved, 2PN fertilization rate, clinical pregnancy rate, abortion rate, and live birth rate between the two groups (*P* > 0.05). When the dominant large follicles' diameter was ≤ 14 mm, the final oocyte maturation rate (92.7% vs. 88.1%, *P* = 0.023), high-quality embryo rate (72.9% vs. 61.8%, *P* = 0.047) and live birth rate (54.5% vs. 31.9%, *P* = 0.043) of Subgroup A1 were significantly higher than those of Subgroup A2. In contrast, when the dominant large follicles' diameter was > 14 mm, no statistical difference was observed in all data.

**Conclusions:**

Large follicle puncture and aspiration in long GnRH-a protocol COH could improve the oocyte maturation rate and high-quality embryo rate in patients with asynchronized follicles. However, clinical pregnancy and live birth rates were not significantly improved. In addition, when the dominant follicles' diameter did not exceed 14 mm, large follicles puncture and aspiration significantly improved the patient's oocyte maturation rate, high-quality embryo rate and live birth rate.

## Introduction

Typically, the human reproductive cycle is characterized by single follicle development and ovulation. During the luteal-follicular transition phase of the natural menstrual cycle, antral follicles sensitive to FSH are recruited for rapid growth due to the elevation of FSH concentrations beyond the FSH threshold window. Afterward, due to the negative feedback induced by inhibin B (INH-B) and estradiol (E_2_), FSH begins to decline, and the FSH window closes below the threshold for follicle selection. Except for the most sensitive follicle to FSH, the other follicles stop developing and become atretic. Therefore, the time interval of the FSH threshold window is crucial for selecting a single dominant follicle from the recruited follicles [1]. To improve the success rate of in vitro fertilization-embryo transfer (IVF-ET), it is usually necessary to perform controlled ovarian hyperstimulation (COH) treatment to counter the process of single follicle development and obtain an acceptable number of oocytes for subsequent fertilization and embryonic transfer. In COH cycles, the use of exogenous Gn artificially increases the FSH concentration and prolongs the FSH threshold window period, allowing the follicles that would have been atretic to keep growing. However, the actual size and FSH threshold of each follicle in the cluster differs. Consequently, asynchronization in follicular development occurs, whereby the use of Gn may amplify this asynchronicity [2]. In patients with asynchronous follicles, by secreting high concentrations of INH-B and E_2_ and through paracrine and autocrine effects, the asynchronous dominant follicles (DF) inhibit the growth and development of adjacent small follicles, which in turn affects oocyte maturation rate and pregnancy rate [3, 4]. Therefore, improving follicular development synchronicity is critical in clinical practice. The key to improving follicular synchronization lies in controlling FSH levels during the luteal-follicular transition and avoiding the early recruitment and development of follicles before Gn use.

Among the numerous COH protocols, the long GnRH-a protocol is considered one of the most classical superovulation protocol. Pituitary down-regulation with GnRH-a can reduce FSH levels during the luteal-follicular transition, and reduce cycle cancellation rate caused by premature LH surge in COH [5]. This assists in synchronizing follicular development and ultimately achieving satisfactory clinical outcomes. For ovarian stimulation protocols lacking luteal FSH control, such as the short protocol and gonadotropin-releasing hormone antagonist (GnRH-ant) protocol, use of oral contraceptives (OC), estrogen, GnRH-ant and other pretreatment methods can control the FSH level during the luteal-follicular transition. Huirne Judith AF et al. [6] compared the effects of antagonist regimen with or without OC pretreatment on the number of oocytes retrieved in IVF-ET patients. They postulated that OC pretreatment could decrease the initial concentration of FSH, thereby improving follicular homogeneity and eventually obtaining more high-quality embryos. However, both Kolibianakis et al. [7] and Rombauts et al. [8] reported adverse effects such as increased Gn dosage, prolonged Gn stimulation time, and increased early abortion rate after OC pretreatment. Fanchin et al. [9, 10] reported that the GnRH-ant protocol with E_2_ pretreatment could improve follicular synchronization and increased the number of available embryos, potentially improving the pregnancy rate. However, E_2_ pretreatment only has an inhibitory effect on FSH, and exposure to high levels of LH may adversely affect the implantation rate by altering endometrial receptivity in patients [11]. As GnRH-ant can rapidly inhibit endogenous Gn secretion, premenstrual injection of GnRH-ant is supposed to prevent luteal FSH elevation and promote consistency in follicular development [2]. However, further cumulative data and research are paramount in determining whether this approach can truly improve IVF outcomes.

The above methods enable FSH-sensitive antral follicles to avoid premature exposure to gradient FSH concentrations during the luteal-follicular transition period in case antral follicles of various sizes appear at the beginning of ovarian stimulation. However, in the actual ovarian stimulation process, considering that each follicle has a different sensitivity to Gn, there are still some patients with asynchronized follicles. During ovarian stimulation, the puncture and aspiration of asynchronized large follicles allow the oocytes and granulosa cells to be aspirated. Thereby the inhibitory effect on adjacent follicles and possible induced endogenous LH surge are elimitated, and the oocyte quality can be enhanced [12]. In this study, the clinical data of 180 patients with asynchronized follicles during GnRH-a protocol COH were analyzed and compared in order to provide a basis for the clinical treatment of patients.

## Material and methods

### Study design and participants

This was a retrospective, single-center cohort study. A total of 180 patients with asynchronous follicles who underwent long GnRH-a protocol therapy in the Reproductive and Genetic Center Affiliated to Shandong University of Traditional Chinese Medicine, from January 2018 to December 2020, were recruited. All patients were followed up for at least 1 year, and the data were extracted from the electronic medical record system. Because this was a retrospective investigation, patients were not asked to participate in the analysis.

#### Inclusion criteria

[1] Regular ovulatory menstrual cycle, usually ranging from 27 to 30 days; [2] The presence of ovaries; [3] No current or past diseases affecting gonadotropin secretion, clearance, or excretion; [4] Body mass index (BMI) ranging from 18 to 30 kg/m^2^; [5] No current hormonal therapy; [6] Adequate ovarian exposure on transvaginal ultrasound scan.

#### Exclusion criteria

[1] Age > 42 years; [2] Diagnosis of congenital or acquired uterine abnormalities (such as uterine malformations, uterine adenomyosis, submucosal fibroids, or intrauterine adhesions); [3] Hydrosalpinx; [4] Chromosomal abnormalities; [5] History of repeated implantation failure (RIF); [6] Large follicles or cysts before Gn initiation.

**Fig. 1:**
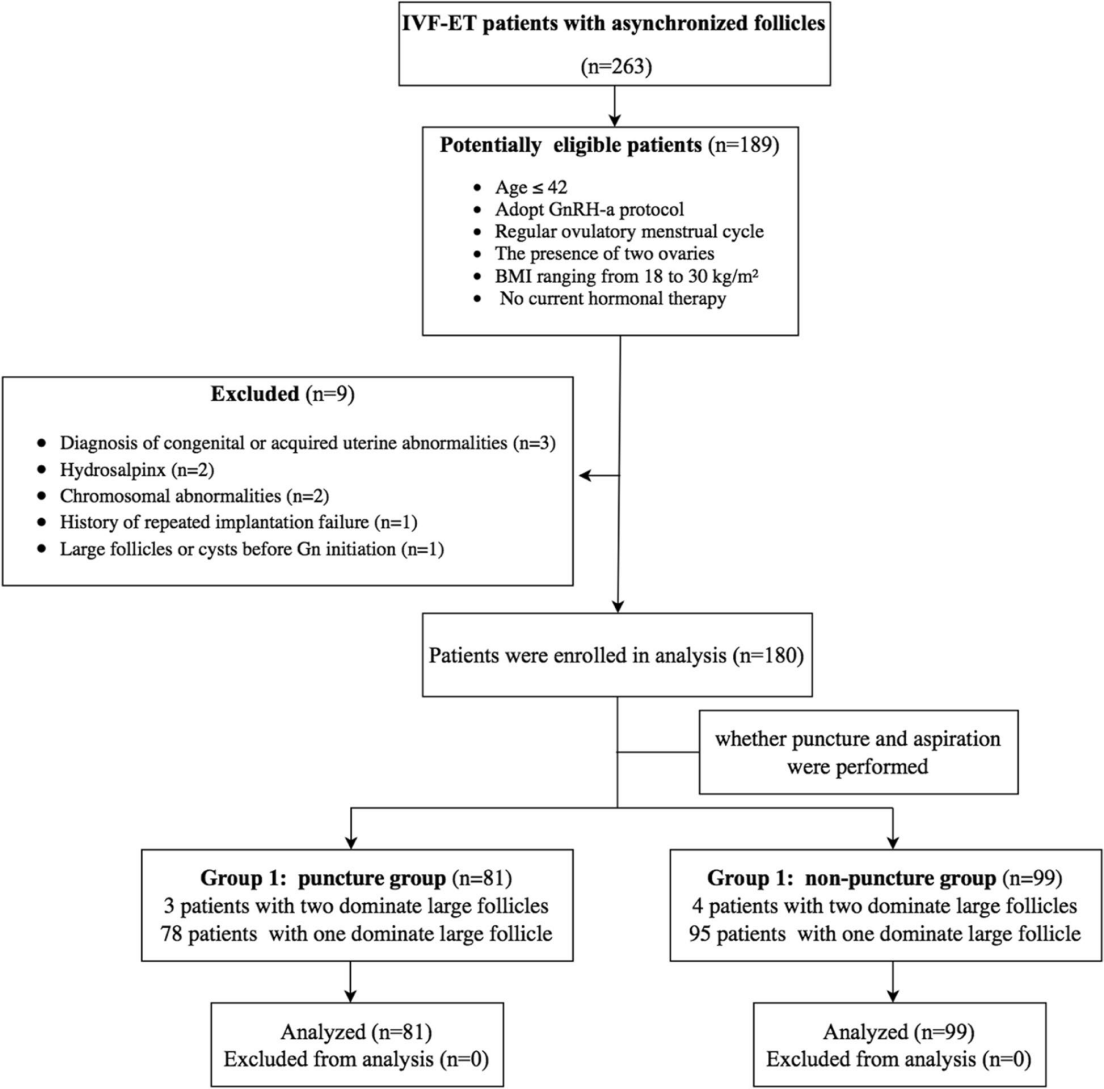
Workflow 1

All patients with asynchronous follicles were divided into either Group 1 (*n* = 81) or Group 2 (*n* = 99), depending on whether large follicle puncture and aspiration were performed or not. Patients in Group 1 underwent transvaginal ultrasound-guided puncture of the large follicles to aspirate the follicular fluid on the day that the dominant large follicles appeared, while patients in Group 2 did not receive the treatment. Medication regimens were similar in both groups (Fig. 1).

**Fig. 2:**
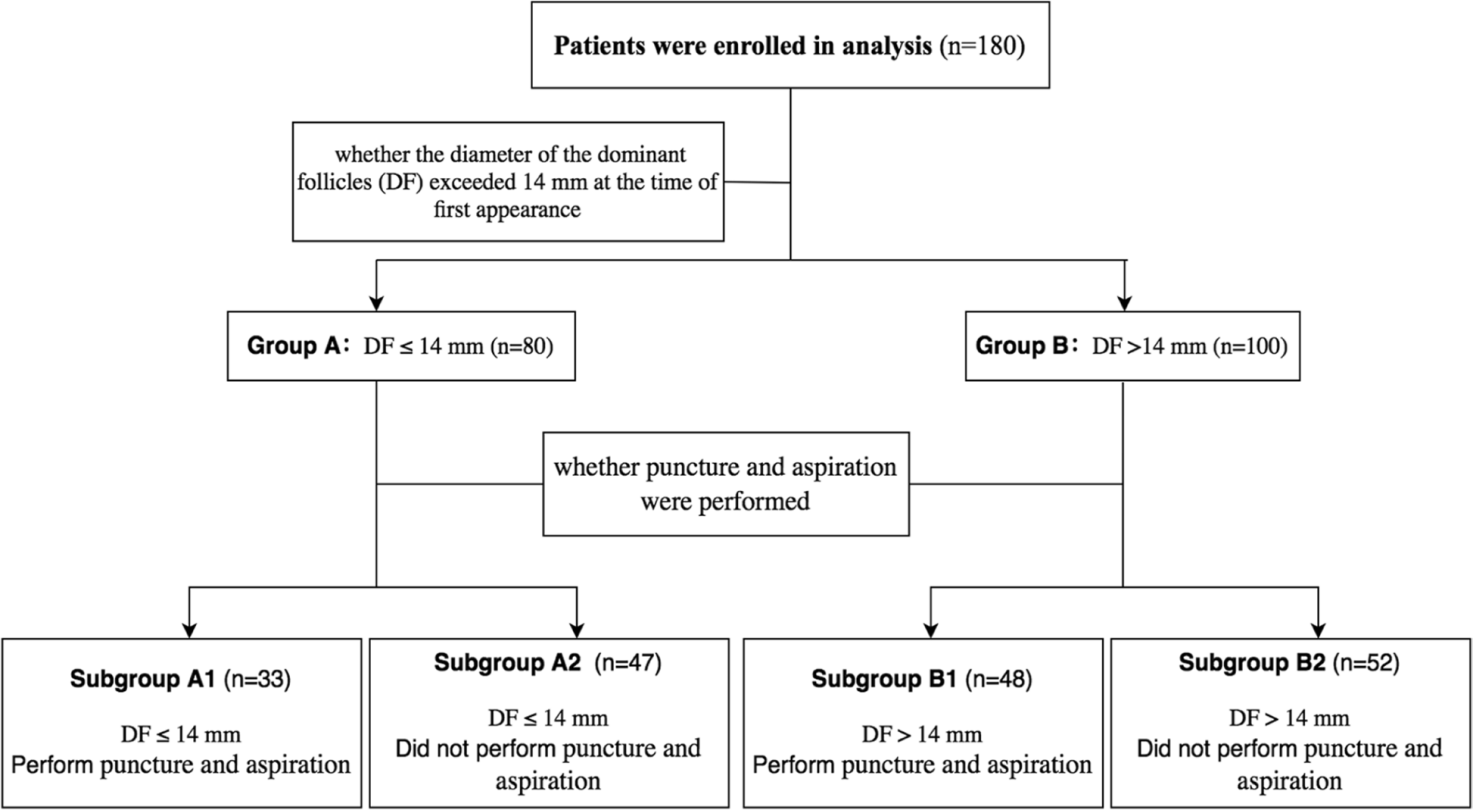
Workflow 2

In addition, we tentatively divided all patients into either Group A (DF ≤ 14 mm) or Group B (DF > 14 mm), if the dominant large follicles’ diameter was less or equal to 14 mm, or exceeded 14 mm at the time of their appearance, respectively. Afterward, depending on whether large follicle puncture and aspiration were performed, the patients were further classified into four subgroups: Subgroup A1 (DF ≤ 14 mm, and patients underwent large follicle puncture), Subgroup A2 (DF ≤ 14 mm, and patients did not undergo large follicle puncture), Subgroup B1 (DF > 14 mm, and patients underwent large follicle puncture), and Subgroup B2 (DF > 14 mm, and patients did not undergo large follicle puncture). This allowed for the comparison of the effects of large follicle puncture and aspiration on the clinical outcomes of patients with asynchronized follicles at different time points (Fig. 2).

### Controlled ovarian stimulation protocol

The option of the stimulation protocols was based on the patients' characteristics, ovarian reserve, and the basal FSH level. All patients received the standard pituitary down-regulation protocol (GnRH-a protocol). They started using either the long-acting preparations of triptorelin acetate (1.0–1.8 mg) or short-acting triptorelin acetate (daily dose 0.05–0.1 mg) for pituitary down-regulation on day 21 of the previous menstrual cycle. After 14–18 days of administration, the pituitary reached the down-regulation criterion. The initial Gn dose (usually 150 to 300 IU per day) was determined based on age, BMI, antral follicle count, basal FSH and E_2_ levels, and previous response to stimulation. The Gn dosage was adjusted every 2–3 days of stimulation. When three or more follicles reached a maximum diameter of 20 mm, human chorionic gonadotropin (hCG, Ovidrel 250 μg; Merck Serono, Darmstadt, Germany) was injected and the oocytes were retrieved 36 h later.

### Large follicle puncture and aspiration

Vaginal ultrasound monitoring was performed regularly after Gn administration. If there were one or two follicles with an average diameter more than 3 mm beyond other small follicles (and the number of other small follicles was > 5) between day 4 and 10 after Gn initiation, we defined these large follicles as the dominant follicles with asynchronous development. The gynecologists either performed large follicle puncture and aspiration on the day the dominant large follicles appeared or not, based on their clinical experience and treatment habits. For patients undergoing large follicle puncture and aspiration, a consent form was first signed. Following this, the asynchronous large follicles were punctured and aspirated with a 17-gauge single-lumen ovum aspiration needle (Cook, Old, Australia) under the guidance of transvaginal ultrasonography. Utmost care was taken to avoid damage to other follicles during the procedure.

### Embryo transfer and luteal phase support

On the third day after oocyte retrieval, the cleavage-stage embryos were graded by morphological criteria based on the number and size of blastomeres and the percentage of fragments [13]. No more than two embryos were transferred (usually one high-quality embryo and one non-high-quality embryo), while the remaining embryos were vitrified. Luteal phase support was provided by vaginal progesterone gel (Crinone, Merck Serono, Germany) 90 mg/day and dydrogesterone (Duphaston, Abbott Laboratories, IL, USA) 20 mg/day. If the patient was successfully impregnated, the luteal phase support would continue until 12 weeks of gestation.

All embryos were vitrified if the patient had a predisposition to ovarian hyperstimulation syndrome (OHSS), an unfavorable endometrium (endometrial thickness ≤ 6 mm or ≥ 16 mm), or progesterone levels ≥ 2 ng/ml on the day of hCG trigger. For frozen embryos transfer, the endometrium was prepared using a natural cycle protocol or an artificial cycle protocol, depending on the patient's condition.

### Results and definitions

The primary outcome was the live birth rate. Secondary outcomes included the oocyte maturation rate, 2pn fertilization rate, high-quality embryo rate, and clinical pregnancy rate. Oocyte maturation rate was defined as the ratio of the number of mature oocytes obtained to the total number of oocytes retrieved [13]. 2pn fertilization was defined as fertilization with two pronuclei on the first day after oocyte retrieval. High-quality embryos were defined as day 3 cleavage-stage embryos with 6–10 cells and embryos with a morphological rating of I and II [14]. Clinical pregnancy was defined as intrauterine pregnancy with at least one positive fetal heartbeat at 6 weeks of gestation or later. Live birth was defined as the delivery of any newborn after 28 weeks of gestation.

### Statistical analysis

All statistical analyses were conducted using IBM SPSS Statistics 26. All statistical analyses were performed with the two-way ANOVA test. A *P*-value < 0.05 was considered statistically significant. Continuous variables were expressed as mean ± SD. The independent samples t-test was used to compare normally distributed data and homogeneity of variance. The nonparametric rank-sum test was used to compare data with non-normal distributions. Categorical data were presented as frequencies and percentages, and differences in the variables were assessed by the chi-square test or the Fisher's exact test.

## Results

Herein, 180 IVF/ICSI patients with asynchronous follicles who underwent the long GnRH-a protocol were included, of which 81 patients underwent large follicle puncture and aspiration (Group 1), whereas 99 patients did not (Group 2). Table 1 summarizes patients’ baseline characteristics of these two groups of patients. There were no significant differences in age, BMI, duration of infertility and basal endocrine level after pituitary down-regulation between the two groups (*P* > 0.05). However, the diameter difference between dominant and secondary follicles (4.46 ± 1.26 vs. 4.03 ± 1.08, *P* = 0.015), Gn usage time (12.57 ± 1.91 vs. 11.89 ± 2.08, *P* = 0.025) and Gn dosage (2321.30 ± 418.04 vs. 2160.83 ± 436.03, *P* = 0.013) were significantly higher in Group 1 than in Group 2.

**Table 1:** Characteristics of patients in Group 1 and Group 2

Variables	Group 1(*n* = 81)	Group 2(*n* = 99)	*P-value*
Age (years)	33.27 ± 4.47	32.27 ± 4.55	0.14
Body mass index (kg/m^2^)	24.03 ± 3.31	23.45 ± 3.23	0.238
Duration of infertility (years)	3.67 ± 2.73	3.47 ± 2.31	0.61
Types of infertility (n, %)			
Primary infertility	29/81 (35.8%)	44/99 (44.4%)	0.24
Secondary infertility	52/81 (64.2%)	55/99 (55.6%)	
Insemination method (n, %)			
IVF	64/81 (79.0%)	77/99 (77.8%)	0.841
ICSI	17/81 (21.0%)	22/99 (22.2%)	
Basal FSH (mIU/ml)	4.36 ± 2.40	4.08 ± 1.94	0.36
Basal LH (mIU/ml)	2.26 ± 1.61	2.24 ± 1.50	0.937
Basal E_2_ (pg/ml)	26.89 ± 12.83	28.06 ± 18.05	0.624
AFC	15.72 ± 6.00	17.20 ± 7.70	0.157
Gn dosage (IU)	2321.3 ± 418.04	2160.8 ± 436.03	0.013
Gn usage time (days)	12.57 ± 1.91	11.89 ± 2.08	0.025
Diameter differences between dominant follicles and secondary follicles (mm)	4.46 ± 1.26	4.03 ± 1.08	0.015

Data are presented as mean ± SD for continuous variables and n (%) for categorical variables. All *P* values were assessed with the use of student’s t-test or χ2

*BMI* body mass index, *FSH* follicle-stimulating hormone, *LH* luteinizing hormone, *E*_*2*_ estradiol, *AFC* antral follicle count

Table 2 illustrates the reproductive outcomes of Group 1 and Group 2. There were no significant differences in the number of oocytes retrieved and the 2PN fertilization rate between the two groups (*P* > 0.05). However, oocyte maturation rate (92.3% vs. 88.9%, *P* = 0.009) and high-quality embryo rate (75.2% vs. 65.7%, *P* = 0.007) were significantly higher in Group 1 than in Group 2. Nevertheless, the final clinical pregnancy rate and live birth rate were not significantly different between the two groups (*P* > 0.05).

**Table 2:** Treatment outcomes in Group 1 and Group 2

Outcomes	Group 1(*n* = 81)	Group 2(*n* = 99)	*P-value*
No. of oocytes retrieved (n)	11.58 ± 6.66	11.19 ± 6.12	0.684
Oocyte maturation rate (n, %)	866/938 (92.3%)	985/1108 (88.9%)	0.009
2PN fertilization rate (n, %)	566/866 (65.4%)	666/985 (67.6%)	0.305
High-quality embryo rate (n, %)	228/303 (75.2%)	247/376 (65.7%)	0.007
Clinical pregnancy rate,CPR (n, %)	46/81 (56.8%)	49/99 (49.5%)	0.329
Pregnancy loss rate, PLR (n, %)	7/46 (15.2%)	9/49 (18.4%)	0.682
Live birth rate, LBR (n, %)	39/81 (48.1%)	40/99 (40.4%)	0.298

Data are presented as mean ± SD for continuous variables and n (%) for categorical variables. All *P* values were assessed with the use of student’s t-test or χ2

Next, we compared the data of the A1 (*n* = 33), A2 (*n* = 47), B1 (*n* = 48) and B2 (*n* = 52) subgroups to investigate whether large follicle puncture and aspiration could affect the clinical outcomes of patients with asynchronous follicles at different time points. Tables 3 and 4 compare the baseline information and treatment outcomes of Subgroup A1 and Subgroup A2. When the diameter of dominant large follicles was ≤ 14 mm, the total days of Gn (13.12 ± 2.06 vs. 11.79 ± 1.77, *P* = 0.003) and the total dosage of Gn (2433.71 ± 359.98 vs. 2246.49 ± 389.04, *P* = 0.032) were significantly greater in Subgroup A1. The oocyte maturation rate (92.7% vs. 88.1%, *P* = 0.023), high-quality embryo rate (72.9% vs. 61.8%, *P* = 0.047), and final live birth rate (54.5% vs. 31.9%, *P* = 0.043) were also significantly higher in Subgroup A1 compared to Subgroup A2.

**Table 3:** Characteristics of patients in Subgroup A1 and Subgroup A2

Variables	Subgroup A1(*n* = 33)	Subgroup A2(*n* = 47)	*P-value*
Age (years)	33.58 ± 3.84	33.02 ± 4.86	0.587
Body mass index (kg/m^2^)	23.57 ± 2.92	23.19 ± 2.54	0.541
Duration of infertility (years)	3.76 ± 3.28	3.21 ± 2.04	0.363
Types of infertility (n, %)			
Primary infertility	11/33 (33.3%)	20/47 (42.6%)	0.405
Secondary infertility	22/33 (66.7%)	27/47 (57.4%)	
Insemination method (n, %)			
IVF	27/33 (81.8%)	39/47 (83.0%)	0.893
ICSI	6/33 (18.2%)	8/47 (17.0%)	
Basal FSH (mIU/ml)	4.66 ± 2.59	3.95 ± 1.60	0.136
Basal LH (mIU/ml)	2.12 ± 1.00	1.93 ± 0.72	0.311
Basal E_2_ (pg/ml)	27.00 ± 11.24	25.34 ± 14.90	0.590
AFC	15.24 ± 5.24	16.74 ± 8.42	0.367
Gn dosage (IU)	2433.71 ± 359.98	2246.49 ± 389.04	0.032
Gn usage time (days)	13.12 ± 2.06	11.79 ± 1.77	0.003
Diameter differences between dominant follicles and secondary follicles (mm)	4.53 ± 1.39	4.03 ± 1.02	0.068

Data are presented as mean ± SD for continuous variables and n (%) for categorical variables. All *P* values were assessed with the use of student’s *t*-test or *χ2*

*BMI* body mass index, *FSH* follicle-stimulating hormone, *LH* luteinizing hormone, *E*_*2*_ estradiol, *AFC* antral follicle count

**Table 4:** Treatment outcomes in Subgroup A1 and Subgroup A2

Outcomes	Subgroup A1(*n* = 33)	Subgroup A2(*n* = 47)	*P-value*
No. of oocytes retrieved (n)	11.21 ± 6.39	10.87 ± 6.17	0.812
Oocyte maturation rate (n, %)	343/370 (92.7%)	450/511 (88.1%)	0.023
2PN fertilization rate (n, %)	208/343 (60.6%)	287/450 (63.8%)	0.366
High-quality embryo rate (n, %)	97/133 (72.9%)	94/152 (61.8%)	0.047
Clinical pregnancy rate, CPR (n, %)	20/33 (60.6%)	20/47 (42.6%)	0.112
Pregnancy loss rate, PLR (n, %)	2/19 (10.5%)	5/21 (23.8%)	0.492
Live birth rate, LBR (n, %)	18/33 (54.5%)	15/47 (31.9%)	0.043

Data are presented as mean ± SD for continuous variables and n (%) for categorical variables. All *P* values were assessed with the use of student’s *t*-test or *χ2*

Tables 5 and 6 compare the baseline information and treatment outcomes of Subgroups B1 and B2. When the diameter of dominant follicles was > 14 mm, there were no significant differences in all comparisons.

**Table 5:** Characteristics of patients in Subgroup B1 and Subgroup B2

Variables	Subgroup B1(*n* = 48)	Subgroup B2(*n* = 52)	*P-value*
Age (years)	33.06 ± 4.88	31.60 ± 4.18	0.109
Body mass index (kg/m^2^)	24.35 ± 3.55	23.68 ± 3.75	0.368
Duration of infertility (years)	3.60 ± 2.31	3.71 ± 2.53	0.826
Types of infertility (n, %)			
Primary infertility	17/48 (35.4%)	24/52 (46.2%)	0.275
Secondary infertility	31/48 (64.6%)	28/52 (53.8%)	
Insemination method (n, %)			
IVF	37/48 (77.1%)	38/52 (73.1%)	0.644
ICSI	11/48 (22.9%)	14/52 (26.9%)	
Basal FSH (mIU/ml)	4.16 ± 2.27	4.19 ± 1.86	0.932
Basal LH (mIU/ml)	2.35 ± 1.92	2.52 ± 1.86	0.655
Basal E_2_ (pg/ml)	26.81 ± 13.93	30.52 ± 20.32	0.294
AFC	16.04 ± 6.48	17.62 ± 7.04	0.249
Gn dosage (IU)	2244.01 ± 440.74	2083.41 ± 464.71	0.080
Gn usage time (days)	12.19 ± 1.72	11.98 ± 2.34	0.618
Diameter differences between dominant follicles and secondary follicles (mm)	4.41 ± 1.17	4.03 ± 1.14	0.106

Data are presented as mean ± SD for continuous variables and n (%) for categorical variables. All *P* values were assessed with the use of student’s *t*-test or χ2

*BMI* body mass index, *FSH* follicle-stimulating hormone, *LH* luteinizing hormone, *E*_*2*_ estradiol, *AFC* antral follicle count

**Table 6:** Treatment outcomes in Subgroup A1 and Subgroup A2

Outcomes	Subgroup B1(*n* = 48)	Subgroup B2(*n* = 52)	*P-value*
No. of oocytes retrieved (n)	11.83 ± 6.89	11.48 ± 6.12	0.787
Oocyte maturation rate (n, %)	523/568 (92.1%)	535/597 (89.6%)	0.146
2PN fertilization rate (n, %)	358/523 (68.5%)	379/535 (70.8%)	0.398
High-quality embryo rate (n, %)	131/170 (77.1%)	153/224 (68.3%)	0.055
Clinical pregnancy rate,CPR (n, %)	26/48 (54.2%)	28/52 (55.8%)	0.872
Pregnancy loss rate, PLR (n, %)	5/27 (18.5%)	4/28 (14.3%)	0.952
Live birth rate, LBR (n, %)	21/48 (43.8%)	25/52 (48.1%)	0.664

Data are presented as mean ± SD for continuous variables and n (%) for categorical variables. All *P* values were assessed with the use of student’s *t*-test or *χ2*

## Discussion

In recent years, assisted reproductive technologies have developed rapidly. In particular, improvements in controlled ovarian stimulation protocols allowed the development of multiple synchronized follicles in the same oocyte retrieval cycle, which helps patients achieve increased high-quality embryo and pregnancy rates. At present, among the numerous COH protocols, the long GnRH-a protocol is considered one of the most classical superovulation protocol. By binding to GnRH receptors, GnRH-a results in a dramatic decrease in the number of receptors on the pituitary surface, whereby the receptors lose response to endogenous and exogenous GnRH. After 14 days of GnRH-a, the pituitary gland reaches a state of down-regulation, avoiding the appearance of early-onset LH surge and promoting the synchronous development of follicles [5]. However, given that the sensitivity to Gn stimulation is not completely consistent during superovulation, growth differences between developing follicles cannot be completely overcome. Therefore, asynchronous follicular development can still be clinically observed in some patients. The determination of the HCG day is complex due to asynchronous follicles and is a challenge regularly faced by doctors. The immature or postmature follicles in patients are frequently collected, which reduces the number of effective embryos and affects the final clinical outcomes.

In the natural cycle, the other follicles on the side of the ovary where the dominant follicle is located are smaller than the contralateral follicle, indicating that the presence of the dominant follicle may inhibit the growth of other non-dominant follicles [15, 16]. Follicular dominance consists of two primary components: indirect endocrine action and direct intra-ovarian regulation. The dominant follicle is able to secrete high concentrations of INH-B and E2, leading to small follicles atresia due to the down-regulation of FSH concentrations. The dominant follicle is more sensitive to FSH stimulation due to the increased number of granulosa cells and the increase of FSH receptors, which allows the dominant follicle to survive despite the later decrease in circulating FSH concentration. This process can be regarded as an indirect endocrine effect of the dominant follicle [3, 17]. In addition to this systemic endocrine factor, the dominant follicle can directly inhibit the growth and development of small follicles through paracrine and autocrine effects. The concentration of insulin-like growth factors-1 (IGF-1) is higher in the dominant follicle than in the small follicles, and the IGF-1 system can amplifie FSH and stimulate the growth of follicles. The presence of a dominant follicle allows increased IGF-binding protein (IGFBP) production on small follicles, thus reducing the concentration of available IGF-1. Therefore, the dominant follicle can continue growing despite reduced FSH concentrations, while the adjacent small follicles undergo follicular atresia and die [18, 19]. Additionally, Spears et al. [20] determined that the dominant follicle's effects were established when the follicles were cultured together, while this phenomenon did not arise when follicular clusters were cultured separately under similar conditions. Therefore, it is hypothesized that there is a direct interaction between the follicles, which allows the dominant follicle to influence the fate of the adjacent follicles by enhancing the endocrine dominance effect.

In clinical applications of the down-regulation protocol to induce superovulation, under the action of a high dose of FSH, the follicles developed in follicular clusters may still appear uneven in size. This phenomenon suggests that even if exogenous FSH can attenuate the indirect endocrine effect, the appearance of large dominant follicles will still affect the growth of the remaining small follicles due to internal regulation in the ovaries, which is, in turn, detrimental to clinical outcomes. In the ovine superovulation model, the dominant follicle influenced the number of follicles and embryos obtained from the ovary on the dominant follicle's side in ewes during high-dose FSH treatment and decreased embryonic viability as well [4]. Semra Kahraman et al. [21] studied the impact of follicular dynamics on early human embryo development for the first time. They discovered that for follicles derived from homogenous cycles, the odds of obtaining top or good quality blastocysts were 1.370-fold higher than that of heterogeneous follicles. Therefore, follicular sizes and variable growth rates in IVF-ET cycle could affect early human embryonic development.

In superovulation cycles, puncture and aspiration were performed to remove oocytes and granulosa cell from the dominant large follicles. The dominant follicles disappeared, thereby eliminating its direct inhibition on the adjacent follicles through interfollicular interactions. After the steroid-rich follicular fluid was aspirated, the steroid hormone level in the circulation decreased. This avoids the asynchronous development of endometrial glandular epithelium and stroma caused by the premature rise of LH surge and progesterone, helping to improve the embryo implantation rate [22, 23]. Fisch et al. [24] reported on a 39-year-old woman with a poor response to repeated IVF, who had prematurely developed 27-mm dominant follicles punctured during superovulation treatment and found that the number of oocytes retrieved increased after the puncture, and a healthy pregnancy was eventually attained. Animal experiments have revealed that removing the dominant follicle in cows 48 h before hyperstimulation could promote follicular growth and increase the number of transferable embryos [25]. Herein, we compared the clinical outcomes of large follicle puncture and aspiration in patients with asynchronous follicles during COH cycles in order to investigate whether large follicle puncture and aspiration were beneficial in clinical treatment.

In this study, compared with Group 2, the differences between the diameter of the dominant and secondary follicles in Group 1 were more significant, and for this reason, clinicians often prefer large follicle puncture and aspiration for such patients. After the removal of the dominant follicles by puncture, the remaining small follicles became the target of Gn medication. Therefore, patients in Group 1 needed longer Gn usage time and Gn dosage to enable small follicles to reach the standard size for the HCG day. Herein, it was determined that dominant follicles tended to be those that developed faster than the remaining adjacent small follicles. However, their development rate did not correspond to the Gn usage days. The short Gn administration time of the dominant follicles can affect the maturation of the cytoplasm. The expression of the nucleus mainly depends on the regulation of cytokines in the cytoplasm, and consequently, the immaturity of the cytoplasm can lead to defects in the quality and quantity of cytokines. Finally, the quality of follicles and embryos are badly affected [26]. In addition, it was challenging to decide the HCG day in Group 2 due to the presence of dominant follicle. On the day of oocyte retrieval, only a few follicles met the diameter criterion for mature follicles. In contrast, the number of small follicles was high, implying that the oocyte maturation rate and high-quality embryo rate were lower than that of Group 1. However, there were no statistically significant differences between the two groups in the 2pn fertilization rate, clinical pregnancy rate, and live birth rate. This may be due to embryo formation and development is a complex and dynamic process. During the whole process of embryonic development, multiple factors, namely, the ultrastructure of oocytes and embryos, embryonic development potential, endometrial receptivity, etc., can affect normal fertilization, embryonic development, and ultimately, clinical pregnancy.

Son WY et al. [27] compared the pregnancy outcomes of IVM cycles in 160 patients and determined that in hCG-primed IVM cycles, ipsilateral immature oocytes were adversely affected when the diameter of dominant follicles exceeded 14 mm, and better clinical outcomes could be achieved by retrieving oocytes before the dominant follicles' diameter exceeded 14 mm. To determine whether large follicle puncture and aspiration affected the outcomes when the dominant follicles appeared at different time points, the 180 patients were tentatively divided into 4 subgroups based on whether the diameter of DF exceeded 14 mm on the day of their appearance and whether large follicle puncture and aspiration were performed. The results revealed that Subgroup A1 had a higher Gn dosage and total Gn usage time compared to Subgroup A2. Moreover, the oocyte maturation rate, high-quality embryo rate, and live birth rate were significantly better in Subgroup A1 than Subgroup A2. However, no significant differences were observed in Subgroup B1 than Subgroup B2. The results demonstrated that when the diameter of the dominant follicles that appeared did not exceed 14 mm, clinicians could perform large follicle puncture and aspiration to improve the pregnancy outcomes. For patients with dominant follicles greater than 14 mm, no special treatment was required, and the hyperovulation protocol could proceed. Large follicle puncture and aspiration did not result in practical benefits for these patients and might instead increase their economic and psychological burdens. However, this results contradict the findings of Son WY. If ipsilateral immature oocytes were adversely affected when the dominant follicles exceeded 14 mm, the large follicle puncture would be more beneficial for such patients. However, our experiment led to the opposite conclusion. Given the paucity of research on this subject and the small sample size included in this experiment, future studies should focus on expanding the sample size to provide more accurate results.

## Conclusions

In conclusion, this study found that there may be patients with asynchronous follicles in GnRH-a long protocol COH. For these patients, large follicle puncture and aspiration can improve oocyte maturation and high-quality embryo rates. However, the corresponding Gn dosage will also increase, and clinical pregnancy and live birth rates may not significantly improve. In addition, if the diameter of the dominant follicles does not exceed 14 mm on the day of its appearance, large follicles puncture and aspiration might have a significant impact on improving the oocyte maturation rate, high-quality embryo rate, and live birth rate. Since the small sample size is the main limitation of this retrospective study, this conclusion needs to be further validated in large sample size RCTs in the future.

## Data Availability

All data generated or analyzed during this study are included in this published article.

## Abbreviations

IVF-ET: In Vitro Fertilization-Embryo Transfer
BMI: Body Mass Index
INH-B: Inhibin B
E_2_: Estradiol
COH: Controlled Oarian Hyperstimulation
GnRH-a: Gonadotropin Releasing Hormone-a
GnRH-ant: Gonadotropin Releasing Hormone antagonist
OC: Oral Contraceptives
DF: Dominant Follicles
